# Gut Microbiota Differences in Infants with Cow-Milk-Induced Allergic Proctocolitis: A Comparative Cross-Sectional Study

**DOI:** 10.3390/children12060734

**Published:** 2025-06-05

**Authors:** Zeliha Haytoglu, Dilek Ozcan, Derya Ufuk Altintas

**Affiliations:** 1 Department of Pediatrics, Cukurova University, 01330 Saricam, Adana, Turkey; 2 Division of Pediatric Allergy and Immunology, Cukurova University, 01330 Saricam, Adana, Turkey; dilekozcan@cu.edu.tr (D.O.); deraytek@cu.edu.tr (D.U.A.)

**Keywords:** cow-milk-induced allergic proctocolitis, faecal microbiota, infants, microbial diversity

## Abstract

***Background*****:** Cow-milk-induced allergic proctocolitis (CMIAP) is a non-IgE-mediated food hypersensitivity that often resolves spontaneously but may predispose infants to IgE-mediated allergies and eosinophilic gastrointestinal disorders. Understanding its pathophysiology is crucial for microbiota-based interventions. ***Methods***: We enrolled 32 exclusively breastfed infants—16 with confirmed cases of CMIAP and 16 age-matched healthy controls. The cohorts were sex-balanced (8 F/8 M), term-born (gestational age ± SD: 40 ± 1.2 vs. 39 ± 1.3 weeks), vaginally delivered, and sampled at a mean age of 2.0 ± 0.44 months (range 1.5–3.0) vs. 2.4 ± 0.66 months (range 1.5–3.5). Faecal samples underwent 16S rRNA gene sequencing on the Illumina NovaSeq platform, with diversity and differential abundance analyses. ***Results***: The maternal dairy intake was similar (total dairy: 250 ± 80 vs. 240 ± 75 mL/day; yoghurt: 2.3 ± 1.0 vs. 2.5 ± 1.2 days/week; *p* = 0.72). Bray–Curtis dissimilarity assessments revealed distinct microbiota in infants with CMIAP. Infants with CMIAP had a lower abundance of *Bifidobacterium* (log_2_FC−2.27; q = 0.022; ANCOM-BC), *Collinsella* (−29.35; padj < 0.0001; DESeq2), and *Limosilactobacillus* (−8.01; padj = 0.0285; DESeq2; q < 0.0001; ANCOM-BC) compared with controls. In contrast, *Hungatella* (+24.99; padj < 0.0001; DESeq2), *Veillonella* (+4.73; padj = 0.0221; DESeq2), *Citrobacter* (+10.44; padj = 0.0124; DESeq2), and *Ruminococcus gnavus* (+2.69; q < 0.0001; ANCOM–BC) were more abundant in the CMIAP group. ***Conclusions:*** Infants with CMIAP exhibit gut dysbiosis, which is characterised by the depletion of beneficial commensals and the enrichment of potential pathogens, independent of maternal dairy intake. Further studies should establish whether these microbiota alterations are causal or consequential in CMIAP.

## 1. Introduction

Allergic proctocolitis (AP) is classified as a non-IgE-mediated food allergy. Most cases of allergic proctocolitis are caused by cow milk proteins, and it is most common in exclusively breastfed infants [1,2]. Cow-milk-induced allergic proctocolitis (CMIAP) is a non-IgE-mediated food hypersensitivity, manifesting in otherwise healthy term infants during the first six months of life in the form of painless rectal bleeding and mucus-streaked stools [1,2]. While symptoms typically resolve by 12–18 months following maternal dairy exclusion, CMIAP confers a twofold increased risk of later IgE-mediated milk or egg allergy and has been linked to eosinophilic gastrointestinal disorders, positioning it as an early event in the atopic march [3,4,5]. The pathophysiology of CMIAP is not well understood. Several mechanisms have been suggested to underlie the development of food-protein-induced gastrointestinal allergy in general. First, antigen delivery, whereby intact cow milk proteins such as β-lactoglobulin and κ-casein resist gastric hydrolysis and appear in breast milk at immunologically active concentrations 4–6 h after maternal ingestion [6]. Second, epithelial barrier immaturity, wherein neonatal gut mucosa exhibits a low expression of tight-junction proteins (e.g., claudin-3 and occludin) and heightened paracellular permeability, facilitating antigen translocation [7]. Concurrently, mucosal immune immaturity—with underdeveloped FoxP3+ regulatory T cells and a Th2-skewed cytokine milieu (e.g., IL-4 and IL-13)—predisposes infants to exaggerated local inflammation [8]. Rectal biopsy specimens from affected infants reveal eosinophilic infiltration and Th2 polarisation, mirroring patterns seen in murine models where germ-free mice colonised with *Enterobacteriaceae* developed more severe proctocolitis than those colonised with *Bifidobacterium* [9].

Emerging evidence implicates the dysbiosis of gut microbiota as a critical fourth driver. The high-resolution sequencing of infants with CMIAP revealed the depletion of *Bifidobacterium* and the enrichment of *Proteobacteria* and *Clostridiaceae*, directly correlating with disease severity and barrier markers in these patients [10]. Cross-sectional studies have reported the consistent depletion of *Bifidobacterium* and the enrichment of *Proteobacteria* (e.g., *Citrobacter*) or *Veillonella* in infants with CMIAP [11,12].

However, understanding the specific attributes of gut microbiota in patients with CMIAP remains nascent. Previous studies conducted in this domain were subject to certain limitations, including the use of participants with inadequate diagnostic criteria for non-IgE-mediated cow’s milk allergy. They examined the microbiota using only culture-based methods [13,14], as well as combining individuals with non-IgE- and IgE-mediated cow’s milk allergies together in the analysis [15,16].

Defining robust microbial biomarkers of CMIAP could (i) facilitate non-invasive early diagnosis, (ii) rationalise targeted probiotic or prebiotic interventions to restore mucosal tolerance, and (iii) prevent unnecessary maternal dietary restrictions that undermine breastfeeding.

Accordingly, we hypothesise that exclusively breastfed infants with challenge-confirmed CMIAP harbour a compositionally distinct faecal microbiota compared to age-matched healthy controls. To test this, we performed a single-centre, cross-sectional 16S rRNA gene sequencing study using high-resolution amplicon-sequence-variant analysis and bias-corrected differential abundance statistics.

## 2. Materials and Methods

### 2.1. Study Design and Participants

This cross-sectional study was conducted between January 2022 and December 2023 at Çukurova University Faculty of Medicine, Balcalı Hospital (Adana, Türkiye), which is a tertiary-care referral centre for paediatric allergy and gastroenterology. Two cohorts were consecutively enrolled.

The CMIAP group (n = 16) comprised term infants with challenge-confirmed cow-milk-induced allergic proctocolitis, recruited from the Paediatric Allergy and Immunology clinic.

The control group (n = 16) comprised age-matched, healthy term infants attending routine baby visits in the General Paediatrics department.

All 32 infants were vaginally delivered, full term (≥37 weeks gestation), and were exclusively breastfed. The eligibility criteria permitted infants up to 6 months of age. Faecal sampling was performed during the index bleeding episode, before the initiation of any maternal dairy restrictions.

All mothers completed a standardised food frequency questionnaire assessing the consumption of dairy products. Maternal dairy intake was assessed using a standardised food frequency questionnaire that captured all commonly consumed milk products—milk, yoghurt, kefir, buttermilk, fermented creams, and a range of cheeses. In the analysis, every specific cheese type (e.g., cottage cheese, mozzarella, cheddar, and ricotta) was collapsed into a single “cheese” subcategory to facilitate comparison across groups.

### 2.2. Eligibility Criteria

Infants were eligible if they met all of the following criteria:

1. Exclusively breastfed, <6 months old;
2. Born at term (≥37 weeks’ gestation), vaginally delivered;
3. No history of diarrhoea or gastrointestinal infection;
4. No autoimmune, atopic, or metabolic disease in infant or mother;
5. No exposure to antibiotics, probiotics, immunosuppressants, or chemotherapeutic agents (infant or mother during pregnancy).

CMIAP was diagnosed on the basis of the following four criteria: (i) painless rectal bleeding with mucus in an otherwise healthy infant; (ii) symptom remission after the maternal elimination of cow’s milk; (iii) symptom recurrence on blinded maternal re-challenge; and (iv) the exclusion of alternative causes of bleeding (e.g., anal fissure, enteric infection, intussusception, and Meckel diverticulum) [2,17,18]. Faecal samples for microbiome analysis were collected once, during an active symptomatic episode.

### 2.3. Faecal Sample Collection

Nappies were delivered to the clinic within 30 min of defaecation. Under aseptic conditions, the attending physician transferred the stool into sterile tubes, which were flash-frozen at −80 °C until DNA extraction.

### 2.4. DNA Extraction and 16S rRNA Amplicon Sequencing

Stool DNA was extracted with the QIAamp Fast DNA Stool Mini Kit (Qiagen, Hilden, Germany; Cat. No. 51604). DNA concentration and quality were measured on both a NanoDrop 2000 (Thermo Fisher Scientific, Waltham, MA, USA) and a Qubit 3 Fluorometer with the dsDNA HS Assay Kit (Thermo Fisher Scientific; Cat. No. Q32851). PCR amplification of the V3–V4 region was performed with the KAPA HiFi HotStart ReadyMix (Roche; Cat. No. KK2602) using custom 16S V3–V4 primers (Oligomer). Amplicons were cleaned using AMPure XP beads (Beckman Coulter; A63880), indexed with the Nextera XT Index Kit v2 (Illumina; FC-131-2001/FC-131-2002), quantified with the KAPA Library Quantification Kit for Illumina (Roche; KK4824), and sized and qualified on a High-Sensitivity D5000 ScreenTape (Agilent; 5067-5592). Libraries were sequenced (2 × 300 bp) on an Illumina NovaSeq 6000 (Illumina; SY-401-1001). All reagents and catalogue numbers are detailed in Table 1. Raw reads have been deposited in the NCBI Sequence Read Archive under BioProject PRJNA1253152 (runs SRR43187075–SRR43187106).

### 2.5. Bioinformatics and Data Quality Control

FASTQ files were processed with Fastp v0.23.2 to trim adapters and low-quality bases [19]. Amplicon sequence variants (ASVs) were inferred using the Divisive Amplicon Denoising Algorithm 2 (DADA2) plugin in QIIME 2 v2022.8 [20]. A naive Bayes classifier trained on SILVA (release 138) sequences trimmed to the V3–V4 region was used for taxonomy assignment [21]. To further minimise contamination risk, reagent blanks and negative control samples were processed in parallel, and any bacterial ASVs present solely in blank controls were removed from downstream analyses.

### 2.6. Ethics Approval

This study was conducted in accordance with the Declaration of Helsinki and was approved by the Çukurova University Ethics Committee (Approval No. 118/22.01.2022). Written informed consent was obtained from the parents or legal guardians of all participants.

## 3. Statistical Analysis

### 3.1. Baseline Characteristics

Categorical variables (sex) were compared with Fisher’s exact test. Continuous variables were first evaluated for normality with the Shapiro–Wilk test. Variables that met the normality assumption (gestational age, maternal dairy intake, and sampling age) were analysed using two-tailed independent-samples Student’s *t*-tests, whereas non-normal variables (e.g., birth-weight *z*-scores) were examined with two-tailed Mann–Whitney *U* tests. All statistical tests were performed in R (v4.2.2) [22]. Effect sizes were calculated as Hedges’ *g* for parametric comparisons and rank-biserial correlation (*r*) for non-parametric comparisons.

### 3.2. Alpha-Diversity

An alpha-rarefaction workflow was run in QIIME 2 (v2022.8). Using the alpha-rarefaction visualiser, the ASV table was subsampled ten times at each read depth. All rarefaction curves plateaued at 2000 reads and Good’s coverage exceeded 98% at this depth. Therefore, 2000 reads was selected as the working rarefaction depth. Six alpha-diversity indices—dominance, Faith’s phylogenetic diversity, Fisher’s α, the Gini index, the Simpson index, and observed features—were calculated in QIIME 2 at a sequencing depth of 2000, before being exported to R (v4.2.2) for statistical testing. Normality was assessed with the Shapiro–Wilk test. The Gini and the Simpson indices were normally distributed; therefore, they were compared between groups with two-tailed Welch *t*-tests. The remaining four metrics violated normality and were analysed with two-tailed Mann–Whitney *U* tests. Effect sizes (Hedges’ *g* for *t*-tests; rank-biserial *r* for Mann–Whitney tests) with 95% confidence intervals were calculated, and all *p*-values were adjusted for multiple comparisons using the Benjamini–Hochberg false-discovery-rate procedure (*q* < 0.05 regarded as significant).

### 3.3. Beta-Diversity

To obtain a robust measure of between-sample community structure, the ASV table was rarefied ten independent times at a uniform depth of 2000 reads per sample—the depth at which all rarefaction curves plateaued and Good’s coverage exceeded 98%. A Bray–Curtis dissimilarity matrix was calculated for each rarefied table, and the ten matrices were averaged in an element-wise manner in order to yield a single, variance-stabilised distance matrix in QIIME 2. This matrix was exported from QIIME 2 and ordinated in R (vegan v 2.6-4) using principal coordinates analysis (PCoA). Group differences in community composition were assessed with PERMANOVA (adonis2, vegan v2.6-4) [23], and the homogeneity of multivariate dispersions was confirmed via the permutational analysis of multivariate dispersion (PERMDISP 2) (vegan v2.6-4). The variance explained by group status (R^2^) was taken as the effect-size statistic. Two-sided *p*-values < 0.05 were considered significant.

### 3.4. Differential Abundance

Two complementary, compositionally aware methods were employed to detect taxa differing between groups—ANCOM-BC (v2.1.2) (with false discovery rate correction) and DESeq2 (v1.36.0) (using the Wald test with Benjamini–Hochberg adjustment). Taxa with FDR-adjusted q or *p* values below 0.05 were considered.

## 4. Results

Thirty-two infants participated in the study—sixteen diagnosed with CMIAP and sixteen healthy controls. The two groups were comparable in terms of baseline characteristics. Each group comprised eight girls and eight boys. The mean gestational age was 40 ± 1.2 weeks in the CMIAP group and 39 ± 1.3 weeks in the control group. All infants were exclusively breastfed and all were delivered vaginally. The mean age at sampling was 2.0 ± 0.44 months (range 1.5–3.0) for infants with CMIAP and 2.4 ± 0.66 months (range 1.5–3.5) for the controls. All infants were born at term with birth weights > 2500 g, and their weight-for-age z-scores—calculated at sampling using the 2006 WHO Child Growth Standards—ranged from –1.5 to +1.3, thereby falling entirely within the normal interval (–2.0 ≤ z ≤ +2.0). In our cohort, the baseline demographic and clinical characteristics of the study infants are summarized in Table 2.

**Table 2:** Baseline demographic and clinical characteristics of the study infants.

Characteristic	CMIAP (n = 16)	Control (n = 16)	*p*-Value
Female sex, n (%)	8 (50%)	8 (50%)	1.00
Gestational age (weeks), mean ± SD	40 ± 1.2	39 ± 1.3	0.08
Vaginal delivery, n (%)	16 (100%)	16 (100%)	—
Exclusively breastfed, n (%)	16 (100%)	16 (100%)	—
Age at sampling (months), mean ± SD (range)	2.0 ± 0.44 (1.5–3.0)	2.4 ± 0.66 (1.5–3.5)	0.15
Total dairy intake of mothers (mL/day)	250 ± 80	240 ± 75	0.72
Yoghurt consumption of mothers (days/week)	2.3 ± 1.0	2.5 ± 1.2	0.72

### 4.1. Comparison of Alpha-Diversity Indices

**Figure 1:**
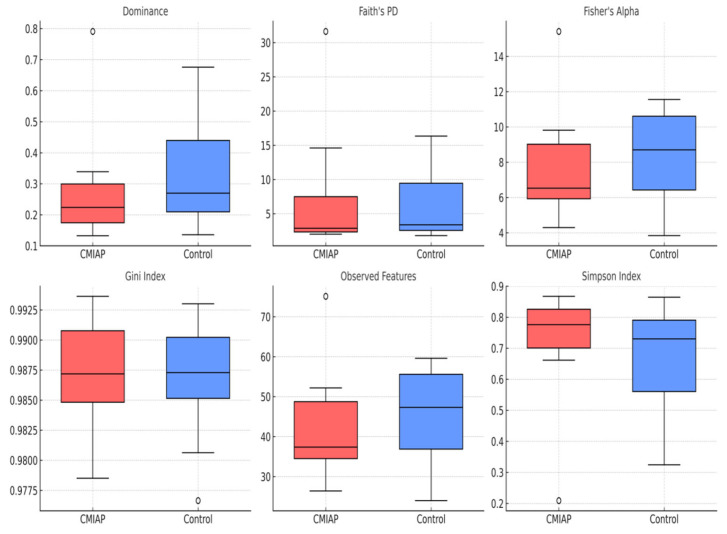
Alpha-diversity indices in stool samples from infants with CMIAP and healthy controls. The box-and-whisker plots show the interquartile range (box), median (horizontal line), and 1.5 × IQR whiskers; individual points represent outliers.

After Benjamini–Hochberg correction, none of the six α-diversity metrics differed significantly between infants with CMIAP and healthy controls (all *q* ≥ 0.46; Table S1). The Berger–Parker dominance index showed the smallest unadjusted *p*-value (*p* = 0.066), yet its FDR-adjusted value remained non-significant (*q* = 0.460). Effect-size estimates were uniformly small to moderate (|*r*| ≤ 0.46; |*g*| ≤ 0.25) and every 95% confidence interval included zero, indicating that within-sample microbial diversity was broadly comparable across the two study groups. Figure 1 demonstrates boxplots of six alpha-diversity indices, comparing infants with CMIAP (red) to healthy controls (blue).

### 4.2. Beta-Diversity Analysis

**Figure 2:**
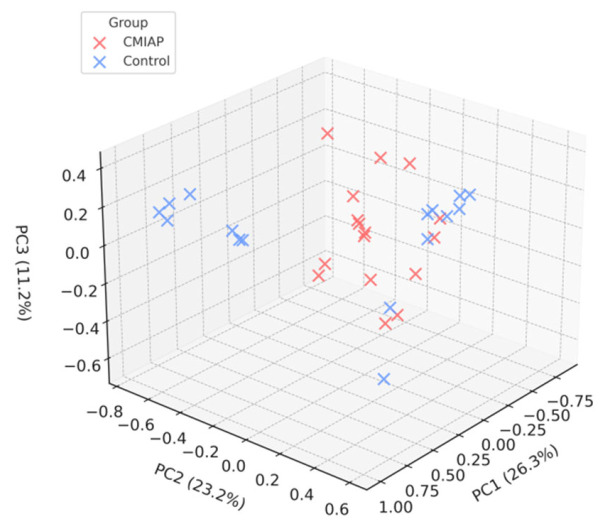
Principal coordinate analysis (PCoA) of the Bray–Curtis dissimilarity matrix for stool samples from infants with CMIAP (red; n = 16) and healthy controls (blue; n = 16). Axes represent the first three principal coordinates (PC1 = 26.3% variance; PC2 = 23.2%; PC3 = 11.2%). Points denote individual infants; grid lines correspond to 0.2 units in the Bray–Curtis space.

The PCoA of the Bray–Curtis matrix showed a partial but visible separation between infants with CMIAP and healthy controls (Figure 2). PERMANOVA indicated that group status accounted for 21% of the total variation in gut microbiota composition (*pseudo-F* = 7.99; R^2^ = 0.21; *p* = 0.001), and multivariate dispersion did not differ between groups (PERMDISP2: *F* = 0.86; *p* = 0.48), confirming that the result was not driven by unequal within-group variability. A post hoc power analysis based on the observed effect size and a sample size of 16 infants per group showed that the study achieved 81% power (micropower; 1000 simulations; α = 0.05), indicating that the design was adequate to detect the observed group effect.

### 4.3. Differential Abundance of Microbial Taxa

Both DESeq2 and ANCOM-BC analyses revealed significant differences in the faecal microbiota of infants with CMIAP compared to healthy controls. DESeq2 identified five genera with differential abundance (Table 3). Notably, strict anaerobes such as *Veillonella* and *Hungatella* were markedly more abundant in the CMIAP group, alongside the *Enterobacteriaceae* genus *Citrobacter*. In contrast, two commensal genera were less abundant in the CMIAP group—*Collinsella* (*Actinobacteriota*) and *Limosilactobacillus* (a *lactobacilli* genus). These taxa were significantly less abundant in infants in the CMIAP group (log_2_FC –29.35 and –8.01, respectively, both adj. *p* < 0.05) (Table 3).

Data in Table 3 are genus-level taxonomic classifications with DESeq2 statistical outputs (Wald test). Positive log_2_ fold changes indicate a higher relative abundance in infants with CMIAP compared to controls, whereas negative values indicate genera that are more abundant in healthy controls.

Table 4 includes the genus-level taxa with statistically significant differential abundance according to ANCOM-BC. Positive log_2_ fold changes indicate that the genus is more abundant in the CMIAP group, whereas negative values indicate a higher abundance in the controls. *p*-values and *q*-values (false-discovery-rate-adjusted *p*-values) from ANCOM-BC’s significance test were reported. All listed taxa were flagged as differentially abundant by ANCOM-BC (diff_abn = TRUE).

Consistent with the DESeq2 findings, ANCOM-BC analysis confirmed key alterations in the CMIAP group (Table 4). Beneficial infant-associated genera were again found to have a lower abundance in infants with CMIAP, whereby *Bifidobacterium* (phylum *Actinobacteriota*) was significantly decreased (log_2_FC –2.27; q = 0.022), corroborating the lower abundance of *Actinobacteria* (*Bifidobacterium*, *Collinsella*) and *Lactobacilli* seen in DESeq2. *Limosilactobacillus* was likewise identified as being less abundant in the CMIAP group (log_2_FC –1.40; q < 0.0001).

Conversely, ANCOM-BC detected an overabundance of certain *Firmicutes* in the CMIAP group. The *Ruminococcus gnavus* group (family *Lachnospiraceae*) was more abundant in the CMIAP group (log_2_FC +2.69; q < 0.0001), which falls in line with the higher abundance of *Hungatella* observed with DESeq2 (both are members of the order *Clostridiales*). *Collinsella* also showed a lower abundance in ANCOM-BC (log_2_FC –2.65; *p* < 0.0001), although this did not reach the 5% false discovery rate threshold (q = 0.097; trend-level significance).

Overall, both methods paint a consistent picture of dysbiosis in CMIAP, which is characterised by the lower abundance of typical infant commensals and the over-representation of opportunistic bacteria.

## 5. Discussion

We observed clear compositional differences in the faecal microbiota of infants with CMIAP compared with age-matched healthy controls. Beneficial commensals were markedly less abundant in CMIAP, including *Bifidobacterium, Limosilactobacillus (a Lactobacillus* genus), and *Collinsella*. Conversely, putative pathobionts were more abundant, including *Hungatella*, *Veillonella*, *Citrobacter* (*Enterobacteriaceae*), and the *Ruminococcus gnavus* group. These shifts were confirmed via two independent pipelines (DESeq2 and ANCOM-BC), underscoring their robustness. Importantly, α-diversity indices did not differ between groups, indicating that CMIAP dysbiosis involves targeted taxonomic imbalances rather than a wholesale loss of diversity.

*Bifidobacterium* and *Limosilactobacillus* dominate the breastfed infant gut, produce short-chain fatty acids, reinforce tight junctions, and expand regulatory T cells; therefore, their reduction compromises barrier integrity and oral tolerance [24,25]. *R. gnavus*—a mucin-degrading anaerobe—has been linked to atopy and inflammatory bowel disease, and longitudinal cohort studies have shown that its expansion precedes allergic manifestations and persists throughout infancy [26,27]. *Veillonella* and *Hungatella* (order *Clostridiales*) thrive when lactate-producing commensals are scarce. Both have been associated with epithelial erosion, and *Veillonella* lipopolysaccharides activate TLR4-mediated inflammation [28,29]. *Citrobacter* (a genus of *Gammaproteobacteria*) and other *Enterobacteriaceae* typically bloom in inflamed niches, amplifying endotoxin-driven responses [30]. *Citrobacter* is an opportunistic pathogen and an elevated presence of *Enterobacteriaceae* is a common hallmark of dysbiosis under inflammatory conditions [30]. Collectively, the loss of immunoregulatory *Bifidobacteria* and *Lactobacilli*, as well as the rise in mucin-degrading or LPS-rich pathobionts, creates a microbiota skewed towards inflammation rather than tolerance, conceivably lowering the threshold for an aberrant immune response to cow milk proteins in CMIAP.

A central question arising from these findings is whether the observed gut dysbiosis is a cause or an effect of CMIAP. Since our study is cross-sectional (snapshot at diagnosis), a temporal sequence cannot be established using this study alone. It is conceivable that dysbiosis in early life predisposes infants to developing allergic proctocolitis. The absence of sufficient tolerogenic bacteria (like *Bifidobacteria*) in the first months of life could impair immune education, making the infant’s gut mucosa hyper-reactive to dietary antigens. Supporting this notion, several longitudinal studies have indicated that gut microbiota imbalances precede and predict allergic outcomes [10,26]. Evidence from the literature strongly suggests that dysbiosis can precede and causatively contribute to CMIAP by creating a permissive environment for inappropriate inflammatory responses to food antigens [31]. On the other hand, dysbiosis may be considered a consequence of the disease process. The intestinal inflammation in CMIAP—however mild or focal—might alter the habitat in ways that favour certain microbes over others. Inflammatory conditions in the gut are known to disrupt microbial ecology by increasing nitrate and oxygen diffusion into the lumen or by releasing host factors that opportunists can exploit. This often results in a bloom of inflammation-associated taxa, particularly *Proteobacteria* [32,33].

Given these considerations, it is likely that the cause and consequence are not mutually exclusive. An initial microbiota imbalance may impair immune tolerance (acting as a cause of CMIAP), and the ensuing allergic inflammation then feeds back to further perturb the microbiota (an effect of CMIAP). This bidirectional interplay could create a self-reinforcing cycle of dysbiosis and inflammation. Breaking this cycle––either by restoring the microbiota or by dampening inflammation––could be key to resolving the condition. Ultimately, only carefully designed longitudinal studies and mechanistic experiments can disentangle the directionality. In the interim, our findings highlight a strong associative link between dysbiosis and CMIAP, which is consistent with the paradigm that the developing gut microbiome is intricately linked to non-IgE-mediated allergic disease pathogenesis. Multi-omics approaches in future studies will complement 16S sequencing and provide a more holistic understanding. Metagenomic sequencing can also identify which genes and pathways are enriched or depleted (e.g., genes for butyrate production might be reduced in CMIAP samples). The metabolomic profiling of the stool could be used to directly measure short-chain fatty acids (SCFAs), lactate, and other metabolites in order to determine how the functional output of the microbiome differs and how that might affect the host (for instance, a drop in butyrate and acetate levels would support the idea of a compromised barrier and regulatory immune tone). The immunological profiling of host responses (such as faecal calprotectin, Th2 cytokine levels, or T-reg cell frequencies in the gut tissue) could be correlated with microbiome data to pinpoint microbiota–immune connections. Such integrated studies would significantly advance our understanding of the microbiome–immune crosstalk in non-IgE-mediated allergy.

The key limitations of our study include the single-time-point sampling and the small sample size. An important additional limitation is the potential influence of the maternal diet on the infant’s gut microbiota. All mothers in our cohort were on unrestricted diets (restriction of dairy products was implemented after diagnosis of CMIAP, and faecal samples were obtained before the restriction; although none reported probiotic/prebiotic supplementation or antibiotic exposure during pregnancy, we did not collect detailed maternal dietary histories).

Maternal exposure to a broad array of macro- and micronutrients can profoundly shape the developing infant’s gut microbiota. However, in the present study, we limited our dietary assessment to the maternal consumption of milk and dairy products and did not evaluate other nutrients or food groups. As a result, the potential influences of maternal macronutrient balance (e.g., total fat, protein, and carbohydrate intake), micronutrient status (vitamins and minerals), and non-dairy fermented foods on infant microbial colonisation were not captured. This narrow focus on dairy intake represents a limitation of our work and underscores the need for future studies to perform comprehensive maternal dietary profiling to fully understand diet–microbiota interactions in early life.

As CMIAP involves dynamic maternal dietary shifts, the timing of sampling can strongly influence microbial readouts. However, by recruiting exclusively breastfed, full-term infants without antibiotic/probiotic exposure, we minimised confounders that often plague infant microbiome studies. Additionally, our use of two differential abundance methods (ANCOM-BC and DESeq2) and multiple alpha-diversity indices improved the robustness of our findings.

## 6. Conclusions

In conclusion, our findings underscore a clear association between gut microbiota dysbiosis and cow-milk-induced allergic proctocolitis in infants. The lower abundance of beneficial microbes and the higher abundance of pathobionts in affected infants suggest that the microbiome likely plays a role in the loss of tolerance and the development of intestinal inflammation. While further research is needed to disentangle the cause and effect, this work contributes to a growing recognition that the microbiota in early life is a critical factor in paediatric allergic diseases. Ultimately, a deeper mechanistic insight could pave the way for novel microbiota-directed strategies to prevent or treat conditions such as CMIAP, promoting immune tolerance in infancy and improving clinical outcomes. The prospect of leveraging the microbiome for therapy is an exciting implication of this research, holding promise for the more effective and tailored management of non-IgE-mediated allergic disorders in the future.

## Figures and Tables

**Table 1:** Reagents and kits used for DNA extraction, quantification, PCR, cleanup, indexing, and sequencing.

Purpose	Product	Supplier (City, Country)	Catalogue No.
DNA extraction	QIAamp Fast DNA Stool Mini Kit	Qiagen (Hilden, Germany)	51604
DNA quantification	Qubit 3 Fluorometer + dsDNA HS Assay Kit	Thermo Fisher Scientific (Waltham, MA, USA)	Q33216/Q32851
PCR amplification	KAPA HiFi HotStart ReadyMix (2×)	Roche (KAPA Biosystems) (Switzerland)	KK2602/KK2601
16S V3–V4 primers	Custom primers	Oligomer (Custom Synthesis)	—
Amplicon cleanup	AMPure XP magnetic beads	Beckman Coulter (Brea, CA, USA)	A63880
Indexing	Nextera XT Index Kit v2 Set A/B	Illumina (San Diego, CA, USA)	FC-131-2001/2002
Library quantification	KAPA Library Quantification Kit for Illumina	Roche (Basel, Switzerland)	KK4824
Size qualification	High-Sensitivity D5000 ScreenTape	Agilent (Santa Clara, CA, USA)	5067-5592
Sequencing	NovaSeq 6000 System	Illumina (San Diego, CA, USA)	SY-401-1001

**Table 3:** Differentially abundant genera identified using DESeq2.

Genus (Phylum)	baseMean	log_2_ Fold Change (CMIAP vs. Control)	*p*-Value	adj. *p*-Value
*Veillonella* (*Firmicutes*)	8485.01	+4.73	0.0016	0.0221
*Hungatella* (*Firmicutes*)	57.24	+24.99	<0.0001	<0.0001
*Citrobacter* (*Proteobacteria*)	303.72	+10.44	0.0007	0.0124
*Collinsella* (*Actinobacteriota*)	202.36	–29.35	<0.0001	<0.0001
*Limosilactobacillus* (*Firmicutes*)	40.75	–8.01	0.0025	0.0285

**Table 4:** Significant taxa detected via ANCOM–BC analysis.

Genus (Phylum)	log_2_ Fold Change (CMIAP vs. Control)	*p*-Value	*q*-Value
*BifidobacteriumActinobacteriota)*	–2.27	0.001	0.022
*(Firmicutes)*	+2.69	<0.0001	<0.0001
*Collinsella (Actinobacteriota)*	–2.65	<0.0001	0.097
*Limosilactobacillus (Firmicutes)*	–1.40	<0.0001	<0.0001

## Data Availability

The raw 16S rRNA sequencing reads generated for this article have been deposited in the NCBI Sequence Read Archive under BioProject PRJNA1253152 (runs SRR43187075–SRR43187106) (https://dataview.ncbi.nlm.nih.gov/object/PRJNA1253152?reviewer=8k9h9hh4ph6b7osi3e41n2fs6t, accessed on 5 June 2025).

## Supplementary Materials

The following supporting information can be downloaded at https://www.mdpi.com/article/10.3390/children12060734/s1. Table S1. Comparison of Alpha-Diversity Metrics Between CMIAP and Control Infants.

## Abbreviations

AP	Allergic proctocolitis
CMIAP	Cow-milk-induced allergic proctocolitis
IgE	Immunoglobulin E
SCFA	Shortchain fatty acid
Treg	Regulatory T cell
IL-10	Interleukin-10
IL-25	Interleukin-25
IL-33	Interleukin-33
TSLP	Thymic stromal lymphopoietin
Th2	T-helper type 2
Th17	T-helper type 17
ASV	Amplicon sequence variant
QIIME 2	Quantitative insights into microbial ecology version 2
ANCOM–BC	Analysis of composition of microbiomes with bias correction
DESeq2	Differential expression analysis for sequence count data
PERMDISP	Permutational analysis of multivariate dispersion
PERMANOVA	Permutational multivariate analysis of variance
PCoA	Principal coordinate analysis
DNA	Deoxyribonucleic acid
PCR	Polymerase chain reaction
IRB	Institutional review board
log_2_FC	Log2 fold change
LPS	Lipopolysaccharide
TLR4	Toll-like receptor 4
FMT	Faecal microbiota transplantation
GOS	Galacto-oligosaccharides
FOS	Fructo-oligosaccharides
SD	Standard deviation
padj	Adjusted *p*-value
lfcSE	Log2 fold change standard error
Wald stat	Wald test statistic
diff_abn	Differentially abundant
FC	Fold Change

## References

[1] Tam J.S. (2020). Food Protein-Induced Proctocolitis and Enteropathy. J. Food Allergy.

[2] Barni S., Mori F., Giovannini M., Liotti L., Mastrorilli C., Pecoraro L., Saretta F., Castagnoli R., Arasi S., Caminiti L. (2023). Allergic Proctocolitis: Literature Review and Proposal of a Diagnostic–Therapeutic Algorithm. Life.

[3] Bahçeci S., Töz P., Çelik F., Can D. (2023). A Different Starting Line for Allergic March: Food Protein-Induced Allergic Proctocolitis. Allergol. Immunopathol..

[4] Nardo D. (2018). Allergic Proctocolitis Is a Risk Factor for Functional Gastrointestinal Disorders in Children. Pediatrics.

[5] Carucci L., Nocerino R., Coppola S., Bedogni G., Capasso P., Giglio V., Canani R. (2025). Factors Influencing the Natural History of Non-IgE-Mediated Gastrointestinal Food Allergies in Paediatric Age: A Prospective Multicentre Cohort Study. BMJ Paediatr. Open.

[6] Matangkasombut P., Padungpak S., Thaloengsok S., Kamchaisatian W., Sasisakulporn C., Jotikasthira W., Benjaponpitak S., Manuyakorn W. (2017). Detection of β-Lactoglobulin in Human Breast Milk 7 Days after Cow Milk Ingestion. Paediatr. Int. Child Health.

[7] Xia Y., Cao H., Zheng J., Chen L. (2022). Claudin-1 Mediated Tight Junction Dysfunction as a Contributor to Atopic March. Front. Immunol..

[8] Nowak-Węgrzyn A., Katz Y., Mehr S.S., Koletzko S. (2015). Non–IgE-Mediated Gastrointestinal Food Allergies. J. Allergy Clin. Immunol. Pract..

[9] Cheng Y., Liu X., Chen F., Rolnik B.M., Chleilat F., Ling Z., Snyder M.P., Zhou X. (2023). The Roles and Mechanisms of Gut Microbiota in Food Allergy. Adv. Gut Microbiome Res..

[10] Martin V.M., Virkud Y.V., Dahan E., Seay H.L., Itzkovits D., Vlamakis H., Xavier R., Shreffler W.G., Yuan Q., Yassour M. (2022). Longitudinal Disease-Associated Gut Microbiome Differences in Infants with Food Protein-Induced Allergic Proctocolitis. Microbiome.

[11] Huang Y.J., Marsland B.J., Bunyavanich S., O’Mahony L., Leung D.Y., Muraro A., Fleisher T.A. (2017). The Microbiome in Allergic Disease: Current Understanding and Future Opportunities. J. Allergy Clin. Immunol..

[12] Berni Canani R., Gilbert J.A., Nagler C.R. (2015). The Role of the Commensal Microbiota in the Regulation of Tolerance to Dietary Allergens. Curr. Opin. Allergy Clin. Immunol..

[13] Nevoral J., Rada V., Vlková E., Bláhová K., Bronský J., Bubáková D., Killer J. (2009). Intestinal Microbiota in Exclusively Breast-Fed Infants with Blood-Streaked Stools. Folia Microbiol..

[14] Kumagai H., Maisawa S., Tanaka M., Takahashi M., Takasago Y., Nishijima A., Watanabe S. (2012). Intestinal Microbiota and Secretory Immunoglobulin A in Feces of Exclusively Breast-Fed Infants with Blood-Streaked Stools. Microbiol. Immunol..

[15] Dong P., Feng J.J., Yan D.Y., Lyu Y.J., Xu X. (2018). Early-Life Gut Microbiome and Cow’s Milk Allergy: A Prospective Case-Control 6-Month Follow-Up Study. Saudi J. Biol. Sci..

[16] Ling Z., Li Z., Liu X., Cheng Y., Luo Y., Tong X., Yuan L., Wang Y., Sun J., Li L. (2014). Altered Fecal Microbiota Composition Associated with Food Allergy in Infants. Appl. Environ. Microbiol..

[17] Mennini M., Fiocchi A., Cafarotti A., Montesano M., Mauro A., Villa M., Di Nardo G. (2020). Food Protein-Induced Allergic Proctocolitis in Infants: Literature Review and Proposal of a Management Protocol. World Allergy Organ. J..

[18] Venter C., Brown T., Meyer R., Walsh J., Shah N., Nowak-Węgrzyn A., Chen T., Fleischer D., Heine R., Levin M. (2017). Better Recognition, Diagnosis, and Management of Non–IgE-Mediated Cow’s Milk Allergy in Infancy: iMAP—An International Interpretation of the MAP (Milk Allergy in Primary Care) Guideline. Clin. Transl. Allergy.

[19] Chen S., Zhou Y., Chen Y., Gu J. (2018). fastp: An Ultra-Fast All-in-One FASTQ Pre-Processor. Bioinformatics.

[20] Callahan B.J., McMurdie P.J., Rosen M.J., Han A.W., Johnson A.J.A., Holmes S.P. (2016). DADA2: High-Resolution Sample Inference from Illumina Amplicon Data. Nat. Methods.

[21] Quast C., Pruesse E., Yilmaz P., Gerken J., Schweer T., Yarza P., Peplies J., Glöckner F.O. (2013). The SILVA Ribosomal RNA Gene Database Project: Improved Data Processing and Web-Based Tools. Nucleic Acids Res..

[22] R Core Team (2023). R: A Language and Environment for Statistical Computing.

[23] Oksanen J., Blanchet F.G., Kindt R., Legendre P., O’hara R.B., Simpson G.L., Solymos P., Stevens M.H., Wagner H. Vegan: Community Ecology Package, R Package Version 2.6-4. https://CRAN.R-project.org/package=vegan.

[24] Moriki D., Francino M.P., Koumpagioti D., Boutopoulou B., Rufián-Henares J.Á., Priftis K.N., Douros K. (2022). The Role of the Gut Microbiome in Cow’s Milk Allergy: A Clinical Approach. Nutrients.

[25] Gavzy S.J., Kensiski A., Lee Z.L., Mongodin E.F., Ma B., Bromberg J.S. (2023). Bifidobacterium mechanisms of immune modulation and tolerance. Gut Microbes.

[26] Chua H.H., Chou H.C., Tung Y.L., Chiang B.L., Liao C.C., Liu H.H., Ni Y.H. (2018). Intestinal dysbiosis featuring abundance of Ruminococcus gnavus associates with allergic diseases in infants. Gastroenterology.

[27] Meadows V., Antonio J.M., Ferraris R.P., Gao N. (2025). Ruminococcus gnavus in the gut: Driver, contributor, or innocent bystander in steatotic liver disease?. FEBS J..

[28] Zhan Z., Liu W., Pan L., Bao Y., Yan Z., Hong L. (2022). Overabundance of Veillonella parvula promotes intestinal inflammation by activating macrophages via the LPS–TLR4 pathway. Cell Death Discov..

[29] Rahman T., Sarwar P.F., Potter C., Comstock S.S., Klepac-Ceraj V. (2023). Role of human-milk-oligosaccharide-metabolising bacteria in the development of atopic dermatitis/eczema. Front. Pediatr..

[30] Hendrickx D.M., An R., Boeren S., Mutte S.K., Lambert J.M., Belzer C. (2023). Assessment of infant outgrowth of cow’s milk allergy in relation to the faecal microbiome and metaproteome. Sci. Rep..

[31] Wurm P., Stampfer L., Greimel T., Leitner E., Zechner E.L., Bauchinger S., Hauer A.C.P., Gorkiewicz G., Högenauer C., Hoffmann K.M. (2023). Gut microbiota dysbiosis in suspected food protein-induced proctocolitis—A prospective comparative cohort trial. J. Pediatr. Gastroenterol. Nutr..

[32] d’Hennezel E., Abubucker S., Murphy L.O., Cullen T.W. (2017). Total lipopolysaccharide from the human gut microbiome silences Toll-like receptor signalling. mSystems.

[33] Litvak Y., Byndloss M.X., Tsolis R.M., Bäumler A.J. (2017). Dysbiotic Proteobacteria Expansion: A Microbial Signature of Epithelial Dysfunction. Curr. Opin. Microbiol..

