# Supplementary material for: Gut Microbiota Differences in Infants with Cow-Milk-Induced Allergic Proctocolitis: A Comparative Cross-Sectional Study
Source: Children (Basel). 2025 Jun 5;12(6):734. doi: 10.3390/children12060734 (PMC12191891; doi:10.3390/children12060734)
Supplement: Supplementary file 1 [file children-12-00734-s001.zip › children-3635748-supplementary.pdf]

Table S1. Comparison of Alpha-Diversity Metrics Between CMIAP and Control Infants.

Metric	Test	*p*	q (BH-FDR)	Effect size ± 95 % CI
Observed features	Mann-Whitney *U*	0.066	0.460	*r* = –0.46 (–0.85 to –0.01)
Dominance	Mann-Whitney *U*	0.322	0.563	*r* = –0.25 (–0.69 to 0.22)
Faith's PD	Mann-Whitney *U*	0.932	0.932	*r* = –0.03 (–0.50 to 0.49)
Fisher's α	Mann-Whitney *U*	0.270	0.746	*r* = –0.28 (–0.75 to 0.23)
Gini index	Welch *t*	0.756	0.882	*g* = 0.14 (–0.65 to 0.93)
Simpson index	Welch t	0.322	0.563	*g*= 0.25 (–0.22 to 0.69)
